# Meeting report: 2009 international conference on molecular neurodegeneration May 18-20, 2009, Xiamen, China

**DOI:** 10.1186/1750-1326-4-43

**Published:** 2009-10-27

**Authors:** Yunwu Zhang, Lisa Owens, Guojun Bu

**Affiliations:** 1 Institute for Biomedical Research and Fujian Provincial Key Laboratory of Neurodegenerative Disease and Aging Research, Xiamen University, Xiamen, China; 2 Burnham Institute for Medical Research, La Jolla, California, USA; 3 Departments of Pediatrics, and Cell Biology and Physiology, Washington University School of Medicine, St. Louis, Missouri, USA

## Abstract

Age-related neurodegenerative diseases are great challenges as the aging population grows. To promote neurodegeneration research and to share recent progress in understanding molecular mechanisms underlying these devastating diseases, the journal *Molecular Neurodegeneration *and Institute for Biomedical Research, Xiamen University co-organized the 2009 International Conference on Molecular Neurodegeneration in Xiamen, China on May 18-20, 2009. The objectives of this meeting were to (1) promote cutting-edge neurodegeneration research in China and in neighboring Asian countries; (2) facilitate the exchange of information relevant to neurodegenerative research; (3) provide education opportunity for students, postdocs and physicians; and (4) provide a platform for investigators at different career levels to interact and network, and to foster collaborations at the international levels. About 100 investigators presented their recent discoveries with a wide range of scopes of neurodegeneration research, including new genes, molecular pathways, animal models, and potential therapeutics.

## Introduction

As life expectancy continues to increase and a growing number of people enter the aged population, neurodegenerative diseases have increasingly become a major problem for which treatments are critically needed. Although much progress has been made in the past 20 years on understanding the mechanisms of neurodegenerative diseases and on developing novel therapeutic strategies, most of these achievements are from the United Stated and European countries. Neurodegeneration research in Asian countries, most of which are very populated and also suffered severely with the spread of neurodegenerative diseases, is lagged far behind. The 2009 International Conference on Molecular Neurodegeneration is designed to bridge this gap. The major aim of this meeting is to provide a unique platform for Asian researchers and scientists from Western countries to exchange information and ideas, to share their most recent research advances, and to establish future collaborations in neurodegeneration studies.

This conference is jointly sponsored by *Molecular Neurodegeneration *, an open access, peer-reviewed online journal that publishes all aspects of neurodegeneration research, and Institute for Biomedical Research, Xiamen University , which is dedicated to revealing the fundamental molecular causes of diseases and devising the innovative therapies of tomorrow. Finacial supports of this meeting come from both foundations and pharmaceutical companies including Alzheimer's Association, Ellison Medical Foundation, National Natural Science Foundation of China, Science and Technology Bureau of Xiamen City, Raptor Pharmaceutical, GlaxoSmithKline, Beckman Coulter, Zeiss, Perkin Elmer, Millipore, Applied Biosystems, and Genetimes Techonolgoy, Inc. Drs. Guojun Bu from Washington University School of Medicine and Huaxi Xu from Burnham Institute for Medical Research, Editors-in-Chief of *Molecular Neurodegeneration*, are co-chairs of this conference.

More than 20 world-renowned scientists, including Dr. Aaron Ciechanover, a 2004 Nobel Laureate in Chemistry for his discovery of the ubiquitin system, and Dr. Steven Heinemann, a member of the US National Academy of Sciences, presented their research progress in the meeting. In addition, researchers from Asian countries, including China (Mainland, Hong Kong, and Taiwan), Japan, and South Korea, also presented their work in short talk and poster sessions.

## List of sessions, chairs, speakers and titles

**Figure 1:**
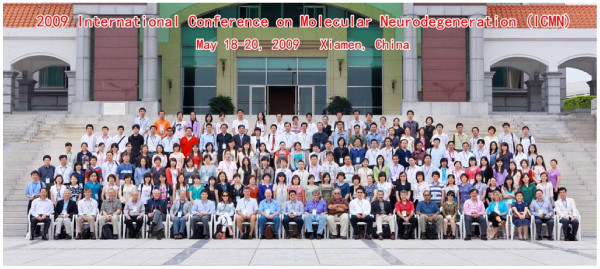
**photograph from the 2009 ICMN**.

See figure 1 for picture.

### Session I & II Chair: Guojun Bu (USA)

• Keynote Speaker: Aaron Ciechanover (Israel Institute of Technology, 2004 Nobel Laureate in Chemistry): "The ubiquitin proteolytic system: from basic mechansisms through human diseases and onto drug targeting"

Session I: ApoE and apoE receptors in AD

• Guojun Bu (Washington University School of Medicine): "ApoE receptors in apoE and Aβ metabolism"

• Mary Jo LaDu (University of Illinois at Chicago): "Aβ42 and apoE structure/function interactions: implications for Alzheimer's disease"

Session II: APP processing: β- and γ-secretases

• Robert Vassar (Northwestern University): "Regulation of BACE1 in Alzheimer's disease"

• Takeshi Iwatsubo (University of Tokyo): "Alzheimer's disease: unraveling the structure-function relationship of gamma-secretase"

• Gopal Thinakaran (University of Chicago): "Function and dysfunction of presenilins"

### Session III & Short Talk Session I Chair: Huaxi Xu (Burnham Institute)

Session III: Presenilin and amyloid beta

• Sam Sisodia (University of Chicago): "Presenilin function in adult neurogenesis"

Short Talk Session I

• Taisuke Tomita (The University of Tokyo): "Identification and analysis of a substrate-specific genetic modulator for the γ-secretase activity"

• Yingying Le (Institute for Nutritional Sciences, Chinese Academy of Sciences): "Activation of β-adrenergic receptor on microglia promotes uptake and clearance of Alzheimer's disease-associated amyloid β peptide"

• Wandong Zhang (National Research Council of Canada): "ABCG2 is up-regulated in Alzheimer's brain with cerebral amyloid angiopathy and acts as a gatekeeper at the blood-brain barrier for Aβ peptides"

### Session IV, V & Short Talk Session II Chair: Sam Sisodia (University of Chicago)

Keynote Address- Steve Heinemann (Salk Institute): "New approaches to understanding Alzheimer's disease"

Session IV: Functions of APP and its processing products

• Hui Zheng (Baylor College of Medicine): "APP function in neurons and synapses"

• Frederic Checler (National Institute of Health and Medical Research, France): "Regulatory functions of APP intracellular domain"

Session V: Parkinson and trinucleotide repeat disorders

• Jie Shen (Harvard Medical School): "Impaired neurotransmitter release in Alzheimer's and Parkinson's diseases"

• Xiaojiang Li (Emory University): "Synaptic toxicity of mutant huntingtin"

Short Talk Session II

• Hongyu Hu (Shanghai Institutes for Biological Sciences, Chinese Academy of Sciences): "Mechanism of α-synulcein aggregation associated with Parkinson's disease"

• Hua Gao (Key Laboratory for Neurodegenerative Disease of the Ministry of Education): "DJ-1 decrease rotenone-mediated dopaminergic cell death via ERK signaling"

• Cristine Alves da Costa (Institut de Pharmacologie Moleculaire et Cellulaire): "DJ-1-mediated cell death control by p53 is regulated by caspase proteolysis"

• Lingqiang Zhu (Department of Pathophysiology and Hubei Provincial Key Laboratory of Neurological Diseases): "Activation of glycogen synthase kinase-3 inhibits long term potentiation with synapse-associated impariments"

### Session VI & Short Talk Session III Chair: Hui Zheng (Baylor College of Medicine)

Session VI: BDNF and novel genes in neurodegeneration

• Eric Tzeng (Beckman Coulter): "The latest total solution characterizes neurodisorder diseases"

Yunwu Zhang (Xiamen University): "A novel gene that inhibits Alzheimer's β-amyloid generation and tau phosphorylation"

Short Talk Session III

• Jin Tae Hong (College of Pharmacy, Chungbuk National University): "Mutant presenilin 2 (N141I) increases beta-secretase activity through increase of ERK pathway dependent hydrogen peroxide generation"

• Ling Li (University of Alabama at Birmingham):"Simvastatin prevents cognitive and hippocampal synaptic deficits in APP/PS1 double transgenic mice"

• Wangxia Wang (University of Kentucky): "MicroRNA miR-107 targets genes related to neurodegenerative disease"

• Qingyan Liu (National Research Council of Canada): "A novel transmembrane protein down regulated in Alzheimer's brains and interacts with a DnaJ-like heat shock protein (DNAJB4)"

• Xiuqi Bao (Institute of Materia Medica, Chinese Academy of Medical Science and Peking Union Medical College): "Establishment of gonadectomy-accelerated brain aging model in mice"

### Session VII & VIII Chair: Xiao-Jiang Li (Emory University)

Session VII: Metal, cell cycle and signaling in neurodegeneration

• Ashley Bush (University of Melbourne): "The metal theory of Alzheimer's disease: from bench to clinic"

• Karl Herrup (Rutgers University): "Cell cycle events as risks for neurodegeneration: a look into mechanism"

• Yong Shen (Sun Health Research Institute): "Tumor necrosis factor and Alzheimer's disease"

Session VIII: AD genetics and therapy

• George Martin (University of Washington): "Classes of gene action with the potential to modulate the times of onset and the rates of progression of neurodegenerative disorders"

• Edward Koo (University of California in San Diego): "Gamma secretase modulators, nonsteroidal anti-inflammatory drugs, and Alzheimer's disease"

• Colin Masters (Mental Health Research Institute of Victoria, Australia): "Aβ oligomers as tractable targets for Alzheimer's disease: diagnostics and therapeutics"

## Competing interests

The authors declare that they have no competing interests.

## Authors' contributions

GB supervised plans for the meeting and made final decisions regarding meeting and planning.

YZ helped prepare and set up for the meeting and wrote this article and cover letter.

LO helped prepare program and agenda for meeting, cooresponded with all involved and served as administrative contact.

